# Quasi-Cubic Magnetite/Silica Core-Shell Nanoparticles as Enhanced MRI Contrast Agents for Cancer Imaging

**DOI:** 10.1371/journal.pone.0021857

**Published:** 2011-07-01

**Authors:** Jos L. Campbell, Jyoti Arora, Simon F. Cowell, Ashish Garg, Peter Eu, Suresh K. Bhargava, Vipul Bansal

**Affiliations:** 1 School of Applied Sciences, RMIT University, Melbourne, Victoria, Australia; 2 School of Medical Sciences, RMIT University, Melbourne, Victoria, Australia; 3 Cell Therapies and Peter MacCallum Cancer Center, East Melbourne, Victoria, Australia; 4 Department of Materials Science and Engineering, Indian Institute of Technology, Kanpur, Uttar Pradesh, India; 5 Cancer Imaging, Peter MacCallum Cancer Centre, East Melbourne, Victoria, Australia; Cornell University, United States of America

## Abstract

Development of magnetic resonance imaging (MRI) contrast agents that can be readily applied for imaging of biological tissues under clinical settings is a challenging task. This is predominantly due to the expectation of an ideal MR agent being able to be synthesized in large quantities, possessing longer shelf life, reasonable biocompatibility, tolerance against its aggregation in biological fluids, and high relaxivity, resulting in better contrast during biological imaging. Although a repertoire of reports address various aforementioned issues, the previously reported results are far from optimal, which necessitates further efforts in this area. In this study, we demonstrate facile large-scale synthesis of sub-100 nm quasi-cubic magnetite and magnetite/silica core-shell (Mag@SiO2) nanoparticles and their applicability as a biocompatible T2 contrast agent for MRI of biological tissues. Our study suggests that silica-coated magnetite nanoparticles reported in this study can potentially act as improved MR contrast agents by addressing a number of aforementioned issues, including longer shelf life and stability in biological fluids. Additionally, our *in vitro* and *in vivo* studies clearly demonstrate the importance of silica coating towards improved applicability of T2 contrast agents for cancer imaging.

## Introduction

Interest in magnetic nanomaterials has persisted over the last few decades primarily due to their applications across many fields such as magnetic data recording, sensing, catalysis and biomedicine [1]–[5]. Magnetic nanomaterials have attracted particular attention in biomedicine due to their great potential in improving the currently available disease diagnostics, prevention, and therapeutic approaches [6]. For instance, the potential of magnetic nanoparticles to precisely deliver highly biotoxic drugs to specific locations in the body [6], as well as their use as highly specialized bio-probes for diagnostic imaging has been demonstrated by attaching biomolecular markers to their surface [1], [7]. With these developments, there is an increasing demand to develop biocompatible magnetic nanomaterials with ultra-sensitive imaging capabilities in order that they can be used for a wide range of *in vivo* medical imaging applications.

Magnetic resonance imaging (MRI) is regarded as a powerful imaging tool because of its high spatial resolution capability, non-invasive nature and its capability to avoid ionizing radiation in contrast to nuclear imaging techniques such as positron emission tomography (PET) [8]–[10]. Briefly, MRI operates by taking advantage of the exceptionally small magnetic moment inherent on each proton that, under the presence of a large magnetic field, produces an effect measurable as a signal on the MR image. The signals produced via T1 relaxation (spin-lattice relaxation) or T2 relaxation (spin-spin relaxation) depends on the sequence parameters programmed to acquire the MR image. Overall, T1 weighted and T2 weighted imaging provide different contrasting effects between fluid and body tissue. For instance T1 weighted images show fluid as dark, water-based tissues as grey and fat-based tissues as bright, thereby very clearly showing the boundaries between different tissues. Conversely, on T2 weighted images, fluid appears bright and water- and fat-based tissues appear grey. The use of contrast agents greatly improves the specificity and sensitivity of MRI by shortening either T1 or T2 relaxation of the water protons adjacent to them, thus providing more detailed information about pathology. Gadolinium-based T1 contrast agents are most commonly used in MRI, however growing concerns over the safety of gadolinium-based contrasts have lead to a major shift towards iron oxide based T2 contrast agents that are deemed to be relatively biologically safe [11]–[13].

Although, iron oxide based contrast agents have been clinically approved for MRI, their use has been predominantly restricted to liver/spleen imaging (AMI-25 Feridex® - not in use anymore) and the gastrointestinal lumen imaging (Lumirem®/Gastromark®). This limitation is primarily due to the larger size of the iron oxide particles involved in these agents, which are either taken up immediately by the reticuloendothelium system after intravenous administration (Feridex®), or are administered orally (Lumirem®/Gastromark®). Therefore, there is a clinical urgency to develop commercially viable and biologically safe contrast agents that can be used for MR imaging of a wide range of body tissues [14]–[16]. Moreover, there have been numerous reports on different synthesis routes to magnetic nanoparticles-based contrast agents, including biologically synthesized magnetic nanoparticles [17]–[18], magnetic nanoparticles with dendrimer cores [19], superparamagnetic liposomes [20], lipid-based MR contrast agents [21], metal-doped magnetic nanoparticles [22]–[25], CoFe2O4@SiO2 particles with fluorescent dyes incorporated [26], and magnetic nanoparticles for both imaging and therapeutic applications [27]. Additionally, in the pre-clinical setting, the trend over the last few years has been towards the development of small (sub-100 nm) iron oxide nanoparticles [24], [28]–[31]. The previous studies suggest that to shift from sub-micron iron oxide particles to their nanoparticulate form in the clinical environment, the challenges that need to be overcome include their low chemical and biological stability, small shelf life, inherent low-to-high cytotoxicity, and low magnetization associated with the iron oxide nanoparticles, which has although been addressed by few recent studies to some extent, it still requires additional efforts in this area [32]–[34]. This is predominantly because the aforementioned properties of MR contrast agents can strongly depend on their synthesis route.

In this manuscript, we address most of the aforementioned issues by demonstrating the development of a T2-weighted, iron oxide-based MRI contrast agent with reasonably low cytotoxicity, high relaxivity, and particularly notable high stability that can be stored at room temperature for more than 6 months without any visible aggregation. The chemical stability of these nanoparticles is achieved by coating them with an inorganic silica (SiO2) layer, leading to Mag@SiO2 core-shell nanoparticles. The resulting nanoparticles were analyzed by a superconducting quantum interference measurement device (SQUID), high resolution transmission electron microscopy (HRTEM), X-ray diffraction (XRD) and a 3 Tesla clinical MRI scanner. Our *in vitro* studies indicate that coating with SiO2 renders these nanoparticles biocompatible and they are actively taken up by prostate cancer cells under *in vitro* conditions. Our preliminary *in vivo* studies with a breast tumor animal model further suggests their potential utility as good MRI contrast agents for tumor imaging.

## Results and Discussion

**Figure 1:**
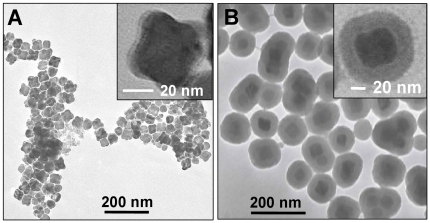
TEM images of (A) Mag and (B) Mag@SiO2 core-shell nanoparticles. Insets show the respective higher resolution TEM images.

Figure 1A shows the TEM image of the magnetic (Mag) nanoparticles, which indicates that the as-synthesized Mag nanoparticles prepared by our synthesis route were quasi-cubic in morphology with good monodispersity and an average size of 40±5 nm. Notably, using our approach, large scale synthesis of Mag nanoparticles could be achieved (at least up to 10 g particles per batch) without compromising the nanoparticle shape or monodispersity. From the higher magnification TEM image, these Mag nanoparticles were found to have spherical edges, and it appears as if these nanoparticles consist of several smaller spherical particles that assemble together giving rise to quasi-cubic structures (inset Figure 1A). It is important to note that under room temperature storage conditions, pristine Mag nanoparticles lose their quasi-cubic morphology and turn spherical after two weeks of synthesis. The shelf life of commercially available MRI contrast agents is in fact one of the major limitations associated with clinical applicability of such materials. SiO_2_ shell coating has been previously demonstrated to provide biocompatibility, particle stability as well as a facile surface for further biofunctionalisation in different nanomaterials [27]–[29]. Therefore, to provide chemical stability to magnetic nanoparticles, a silica shell was grown around quasi-cubic Mag particles (within 3 days of their synthesis), thereby producing Mag@SiO2 core-shell nanoparticles (Figure 1B). The controlled silica coating of Mag nanoparticles led to formation of Mag@SiO2 core-shell structures with a ca. 20±2 nm silica shell around 40±5 nm quasi-cubic Mag nanoparticles (Figure 1B and inset). Large area TEM analysis of Mag@SiO2 core-shell structures indicated that most of the Mag nanoparticles retained their quasi-cubic morphology after silica coating, and more than ca. 75% of particles in the sample were found to be individually coated with a silica shell. However, less than ca. 25% of structures consisted of either two or three or no Mag particles within the silica shell. Notably, this type of particle distribution is typical of a chemical synthesis route, which is not necessarily always explicitly acknowledged in the prevailing literature. Additionally, we observed that after coating Mag nanoparticles with silica, the Mag@SiO2 particles remain stable in phosphate buffer saline (PBS) solution for at least up to 1 mg/mL concentration, as well as in the readily-dispersible powder form for at least up to 6 months. The TEM image shown in Figure 1B was acquired after 6 months of storage of Mag@SiO2 nanoparticles at room temperature and was similar to those imaged immediately after synthesis. This suggests that a silica coating over Mag nanoparticles can significantly improve their stability for long-term storage conditions, thus retaining their magnetic properties by improving their shelf life. This is one of the crucial parameters for developing MRI-based contrast agents for clinical and commercial applications.

**Figure 2:**
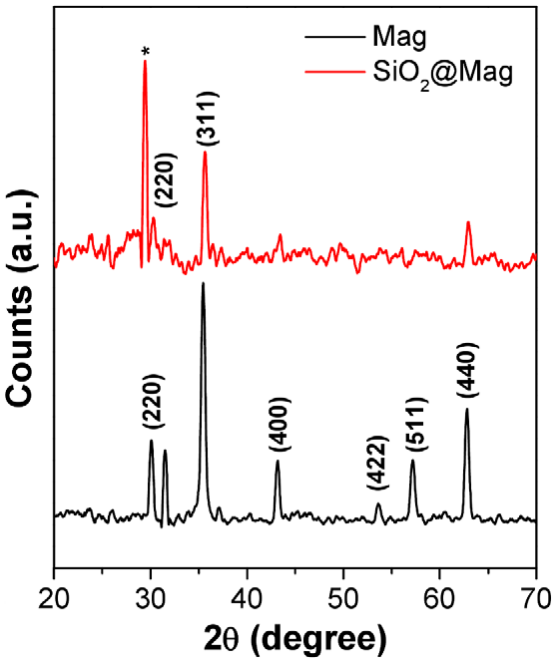
XRD patterns obtained from Mag and Mag@SiO2 nanoparticles. XRD peaks with corresponding Bragg reflections of magnetite have been indicated. (*) corresponds to the XRD peak arising from a mixed Fe-Si phase.

Figure 2 shows the XRD patterns of Mag and Mag@SiO2 nanoparticles. The XRD pattern obtained from quasi-cubic Mag nanoparticles (curve 1) could be indexed based on standard diffraction pattern typically arising from magnetite (Fe_3_O_4_) with major peaks indexed (JCPDS file No 75–0449). After silica coating, most of the diffraction peaks arising from Mag nanoparticles could still be detected. However interestingly, after silica coating, an additional peak at ca. 29.3° 2θ was observed that could be assigned to the (220) plane of a FeSi_2_ phase (curve 2) (JSPDS file no. 73-0963). The mixed Fe-Si phase is most likely formed at the interface of silica and magnetite during core-shell synthesis of Mag@SiO2 nanoparticles.

**Figure 3:**
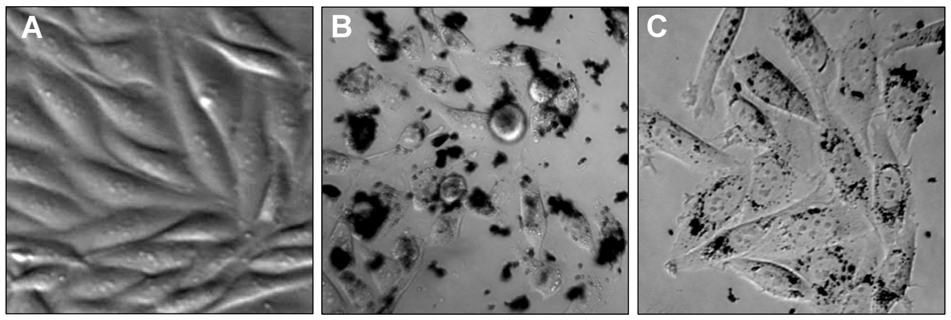
Magnetic hysteresis curve of Mag@SiO2 nanoparticles used for MR imaging of tumor cells and tissues.

High saturation magnetization of MR contrast agents is an important requirement for the magnetic nanoparticles to be used for MRI application. The magnetic hysteresis curve of Mag@SiO2 nanoparticles obtained by SQUID measurement is shown in Figure 3, which was found to have no coercive fields, thus confirming their superparamagnetic nature. Mag@SiO2 nanoparticles were found to possess a relatively high mass magnetization value of 74.4 emu/g, which is comparable to the previously reported mass magnetisation values of 72.9 emu/g for commercially available Resovist iron oxide particles [35].

**Figure 4:**
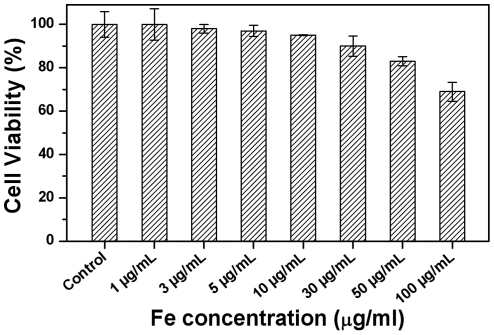
Optical microscopy images of PC3 human prostate cancer cells (control) grown for 24 h (A) in the absence of nanoparticles, and in the presence of (B) Mag and (C) Mag@SiO2 nanoparticles followed by three washings with PBS.

The Mag and Mag@SiO2 synthesized in this study were further tested for their ability to be internalised by human prostate cancer PC3 cells (Figure 4). When subjected to cell uptake studies for 24 h, 50 µg/mL Mag@SiO2 nanoparticles were found to be uptaken by PC3 prostate cancer cells more efficiently than similar a concentration of bare Mag nanoparticles (compare Figures 4B and C). When PC3 cancer cells were exposed to Mag nanoparticles, we observed that bare Mag nanoparticles without any SiO_2_ coating tended to form large aggregates (of dimensions similar to cell size) in the solution over a 24 h exposure period, which restricted their ability to be uptaken by PC3 cells (Figure 4B). As can be inferred from Figure 4B, these large clusters of bare Mag nanoparticles predominantly attach to the exterior of the cells, and are difficult to be internalized by PC3 prostate cancer cells. Conversely, after SiO_2_ coating, Mag@SiO2 nanoparticles remain well-dispersed in the solution even after 24 h, which facilitates their efficient uptake by PC3 cells, as can be seen from a higher density of Mag@SiO2 nanoparticles inside PC3 prostate cancer cells (Figure 4C). Our group and others have previously demonstrated that nanoparticle size and aggregation in biological media can play a crucial role in cellular uptake processes, as non-specific uptake of sub-100 nm nanoparticles is generally observed via endocytosis mechanism of the cells [36]–[39]. Aggregation of bare (pristine) Mag nanoparticles in biological media, and avoidance of their aggregation after silica coating clearly suggests the important role of SiO_2_ coating, and advantage of Mag@SiO2 core-shell nanoparticles over bare Mag nanoparticles for biological applications. Based on results from cell uptake studies, pristine Mag nanoparticles were found to be unsuitable for biological applications, and therefore only Mag@SiO2 nanoparticles were chosen for further studies regarding their suitability for MRI applications.

**Figure 5:**
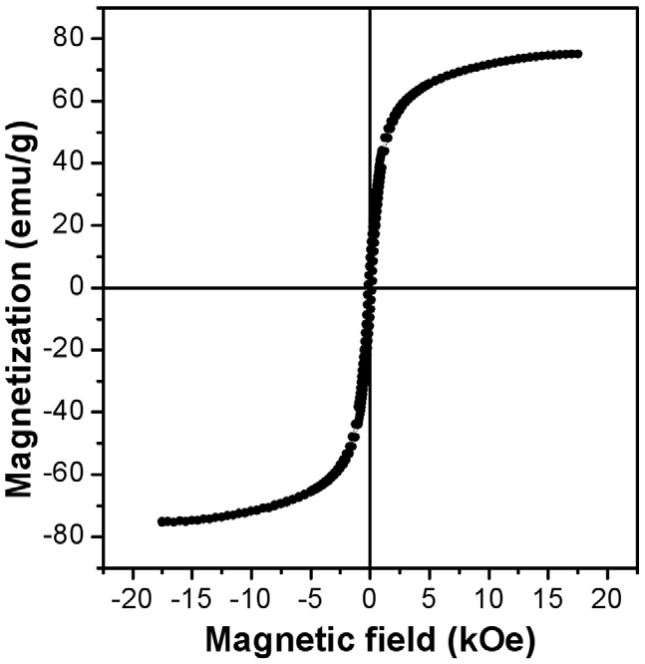
Biocompatibility of Mag@SiO2 nanoparticles assessed using MTT assay after their exposure to PC3 cancer cells for 24 h with respect to different Fe concentration in Mag@SiO2 nanoparticles.

From the cell uptake studies, it is also evident that Mag@SiO2 nanoparticles do not cause any significant change to the morphology of PC3 prostate cancer cells. Previous studies indicate that iron oxide nanoparticles are non-toxic at lower concentration, but can be mildly toxic at higher concentrations [40]–[41]. Before exploring Mag@SiO2 nanoparticles for MRI application, biocompatibility profile of these particles was assessed by performing MTS-based *in vitro* cytotoxicity experiments on PC3 prostate cancer cells, which is one of the measures of biocompatibility (Figure 5). It is evident from Figure 5 that Mag@SiO2 nanoparticles did not significantly affect PC3 cell viability for at least up to 50 µg mL-1 Fe concentrations, at which more than 85% PC3 cells viability was maintained. However further increase in Mag@SiO2 nanoparticles concentration equivalent to 100 µg mL^−1^ Fe resulted in a cell viability loss of ca. 30%. This suggests that Mag@SiO2 nanoparticles reported in this study may be suitable for MRI applications within 50 µg mL^−1^ Fe concentration range. However, this aspect may require further detailed investigation, wherein effect of Mag@SiO2 nanoparticles on cytokine production profile of cells will need to be investigated.

Since magnetic nanomaterials can modulate MR signal enhancement effects, the capability of Mag@SiO2 nanoparticles as T2 MR contrast agent was further assessed in terms of their relaxivity (R2 or relaxation rate, which equals 1/T2 where T2 is spin-spin relaxation time) on a 3 Tesla clinical MRI scanner at an echo time (TE) of 10.86 ms. Relaxivity is a measure of the efficiency of a MR contrast agent to enhance the proton relaxation and increase the efficiency to which image contrast is produced during MRI [42]. The relaxivity measurements were performed both on nanoparticles as suspension in phantoms as well as after being uptaken by PC3 prostate cancer cells. Mag@SiO2 nanoparticles were found to have a high relaxivity value of 263.23 l/mmol/s in cell free suspensions, and 230.90 l/mmol/s for Mag@SiO2 nanoparticles within the PC3 cells. High relaxivity value (that is, better MR contrast) along with high mass magnetisation value for MRI are important considerations when developing T2 contrast agents, as the spin-spin relaxation process of protons in water molecules surrounding the nanoparticles is facilitated by the large magnitude of magnetic spins in nanoparticles [43]–[44]. Mag@SiO2 nanoparticles with high mass magnetization and high relaxivity values may therefore result in strong T2-weighted MR signal intensity decrease as measured by MRI [45]. This is critical in allowing nanomolar activity of contrast agents, which will facilitate in reducing the overall contrast agent dose to the patients.

The relaxivity data also suggests a reduction in the relaxivity value of Mag@SiO2 nanoparticles in PC3 cells after cellular uptake compared with that in suspension. This finding corroborates well with previous studies, which showed that the relaxivities of native iron oxide nanoparticles were higher compared to those after accumulation in the cells [46]–[47]. The mechanisms responsible for this effect have not yet been fully understood, however it can possibly be attributed to the confinement of nanoparticles within endosomes of the target cells, which might cause a build-up of magnetic field inhomogeneities after sub-cellular compartmentalization, which would conversely be absent in uniformly distributed nanoparticles in suspensions [48]. Additionally, the different geometrical arrangement of nanoparticles in suspensions and in cells, and possibly antiferromagnetic coupling as a result of clustering within the sub-cellular compartments may play some role in reducing relaxivity values after cellular uptake [28], [48]. Notably, in contrast to relaxivity values of 230–269 l/mmol/s observed for Mag@SiO2 nanoparticles in this study, commercial Resovist based nanoparticles have been reported with lower values of 151 l/mmol/s [35]. The observed relaxivity value of Mag@SiO2 nanoparticles prepared in this study is also relatively higher than those reported for undoped magnetite particles (218 l/mmol/s) in recent detailed studies [24]. For doped magnetic particles, it has been reported that high relaxivities of up to 358 l/mmol/s can be achieved by doping magnetite with Mn (MnFe_2_O_4_) [24]. However, potential leaching of Mn during administration of these MR contrast agents in the body might pose cytotoxicity issues, and to the best of authors' knowledge, undoped Mag@SiO2 nanoparticles with such high relaxivity values have not hitherto been reported.

**Figure 6:**
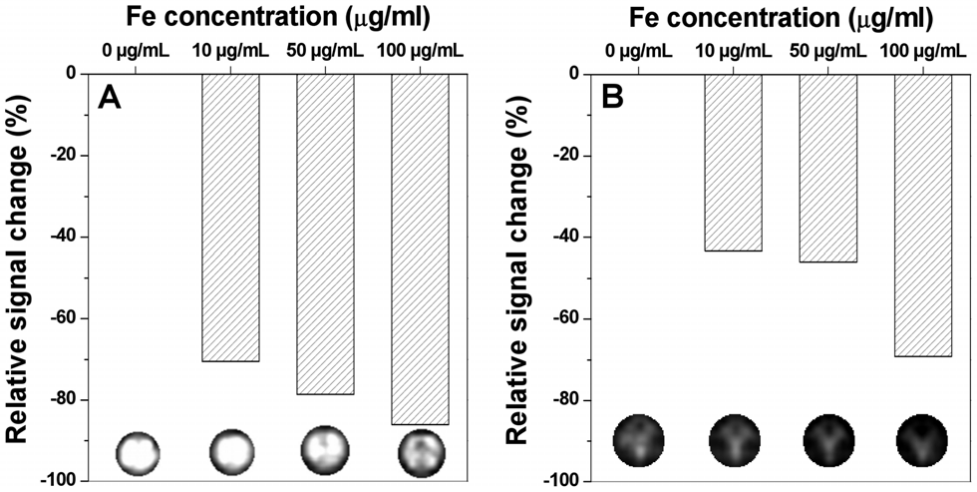
Evaluation of Mag@SiO2 nanoparticles as a T2 MR contrast agent is shown in the form of % signal enhancement with increasing concentration of Fe using a 3 Tesla MR scanner. Panel A shows the studies performed in phantoms for particles in suspension, while panel B shows the similar studies in PC3 human prostate cancer cells after nanoparticles uptake for 24 h. Corresponding T2-weighted MR images of different samples, showing the image darkening effect with increasing Fe concentration are also shown under each bar.

Furthermore, relaxivity studies as a function of different concentrations of Fe in Mag@SiO2 nanoparticles, both as a nanoparticle suspension in phantoms (Figure 6A), and after 24 h of nanoparticle uptake by PC3 prostate cancer cells (Figure 6B) revealed that Mag@SiO2 nanoparticles act as outstanding T2-weighted contrast agents. This is shown by an image darkening effect, demonstrated by drop in R2 (ΔR2/R2_control_) signal intensity with increasing Fe concentrations. For instance, at 100 µg/mL Fe concentration, Mag@SiO2 nanoparticles provide a signal enhancement of ∼90% in comparison to more than 70% signal enhancement during imaging of PC3 prostate cancer cells. This is a significant signal enhancement in comparison to most of the previously reported materials, in which generally only 15–20% signal enhancement has been observed [28]. Such strong MR signal enhancement is expected from Mag@SiO2 nanoparticles because of their relatively high relaxivity and saturation magnetization values.

**Figure 7:**
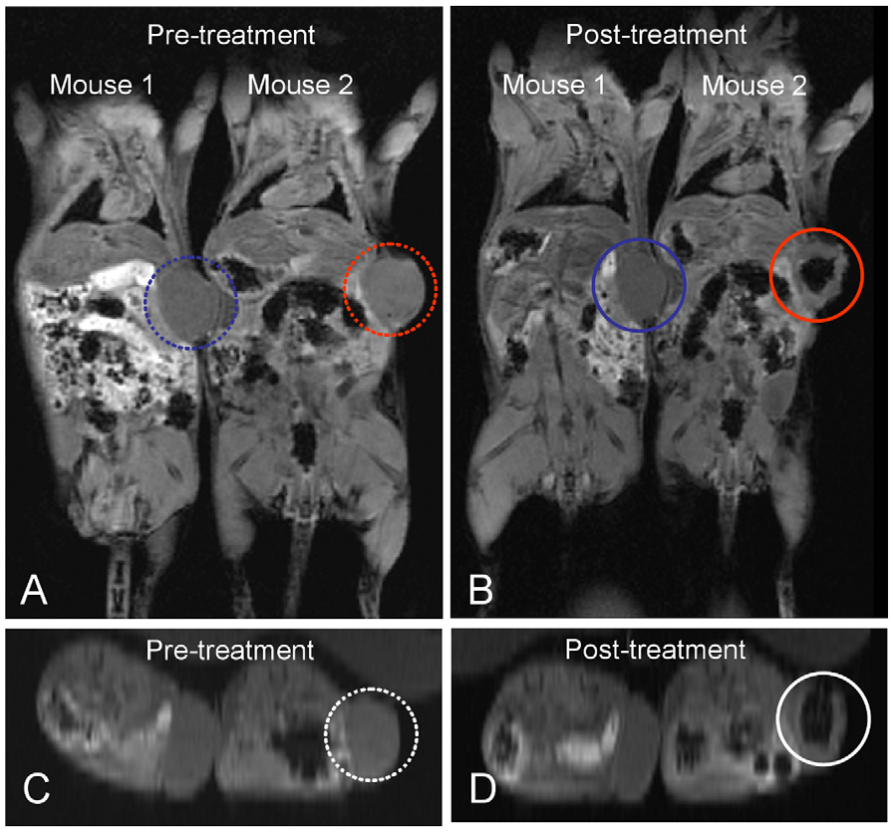
T2-weighted MR images of nude mice with breast tumor obtained (A) before and (B) after injection of MR contrast agent, obtained using a 3 Tesla MR scanner. Mouse 2 was injected with Mag@SiO2 nanoparticles as T2 contrast agent, while Mouse 1 was injected with saline as a control. Tumor sites in the control (mouse 1) and in the treated mouse (mouse 2) have been labelled as blue and red circles respectively. Panels C and D show the higher magnification transverse section images of tumor site corresponding to Panels A and B respectively, wherein tumor region injected with MR contrast agent has been highlighted using white circles.

*In vivo* MRI studies in a breast tumor mouse model also demonstrated T2 signal enhancement at the tumor site by Mag@SiO2 nanoparticles (Figure 7). The images following *in vivo* administration of 10 µg dose of Mag@SiO2 nanoparticles demonstrate its ability to produce MR enhancement of the tumor site relative to the body. T2-weighted signal enhancement effects by the Mag@SiO2 nanoparticles on an MR image are visualised as darkening or contrast between areas infiltrated with Mag@SiO2 nanoparticle and those without nanoparticles. Future studies on Mag@SiO2 can be tailored for targeted MRI, utilising its superior magnetic characteristics in the diagnosis of pathologies.

In summary, important considerations for an efficient MRI contrast agent include smaller particle size, their efficient uptake by cells, reduced aggregation in biological fluids, improved shelf life, and improved biocompatibility. A control over all these parameters will provide an ability to target a range of molecular/cellular imaging applications without causing acute toxicity to the normal cells. Particularly for tumor imaging applications, sub-100 nm particles can provide significant an advantage, as the cut-off diameter of tumor vessel pores is 400–600 nm [41]–[43], [49]–[51].

In this study, we have demonstrated a facile, large-scale synthesis of quasi-cubic magnetite and Mag@SiO2 nanoparticles of sub-100 nm size. The Mag@SiO2 nanoparticles reported here have a shelf life of more than 6 months, and they are efficiently uptaken by the cells without causing significant aggregation or cellular toxicity. The biological half-life of smaller and silica-coated iron oxide nanoparticles is expected to be further increased due to their reduced interaction with the body fluids. This study therefore clearly underlines the importance of SiO_2_ coating towards improving the uptake of Mag@SiO2 nanoparticles by PC3 prostate cancer cells, and improving the shelf life of MR contrast agents. The magnetic-silica composite nanoparticles act as promising T2 contrast agents offering a potentially viable option as a commercial MR contrast agent. This is attributable to their small size, high MR signal enhancement, relative biocompatibility, longer shelf life, and highly modifiable silica surface chemistry which will allow the adhesion of multiple molecular markers for targeted MRI in the future. These characteristics of a T2 contrast agent are highly desirable for magnetic resonance imaging applications at the pre-clinical level and for later use clinically.

## Materials and Methods

### Ethics Statement

The breast tumor mice model was developed in-house, and all the studies involving animals were pre-approved by institutional animal ethics committee.

### Materials

All chemicals were purchased from Sigma-Aldrich and used as received without further modification. The prostate cancer cells (PC3 cell line) were purchased from American Type Culture Collection (ATCC). CellTiter 96 Aqueous One Solution Cell Proliferation Assay (Promega) kit was purchased from Promega Corporation.

### Synthesis of iron oxide nanoparticles

Quasi-cubic iron oxide nanoparticles (referred as ‘Mag’) were synthesized using a two step process significantly modified from Park *et al*, thus leading to controlled large-scale synthesis [52]. During synthesis, an iron oleate complex was first formed by dissolving 5.4 g of iron chloride and 18.25 g of sodium oleate in a solution comprised of 40 mL ethanol, 30 mL distilled water and 70 mL hexane. Once homogenized, the solution was refluxed at 70°C for 4 h, followed by separation of the upper organic layer using a separatory funnel, washing and evaporating off hexane, thereby leaving a waxy iron oleate complex. The iron oxide nanocrystals were formed by dissolving 9.0 g of the iron oleate complex in 1.425 g of oleic acid and 63.3 mL of 1-octadecene, followed by reflux under nitrogen until it reached 320°C, at which point the temperature was held for 30 min and then allowed to cool to room temperature. 250 mL of ethanol was then added to the solution and the magnetite particles were separated via centrifugation, followed by three washing cycles with ethanol. Notably, by designing this protocol, scale up of at least up to 10 g magnetic nanoparticles per reaction could be easily achieved under laboratory conditions.

### Synthesis of silica-coated iron oxide (Mag@SiO2) nanoparticles

Silica-coated iron oxide nanoparticles (Mag@SiO2) were prepared using a method significantly modified from Fang *et al* and Morel *et al* [53]–[54], wherein controlled hydrolysis of silica precursor in the presence of magnetite nanoparticles was performed. In our approach, pre-formed magnetic particles were used as nucleating sites for subsequent hydrolysis of silica precursor around them. Briefly, 1 mg of iron oxide nanoparticles prepared in the previous step were sonicated in a solution consisting 15 mL ethanol and 2 mL deionized water (MilliQ). 1 mL of ammonia (25% solution) was added to the above solution while immersed in a sonicator programmed to switch on for 1 min in every 10 min. Further, an overhead stirrer was additionally used to mix the solution while 4 mL of 1∶60 (tetraethyl orthosilicate∶ethanol) was added at the rate of 0.4 mL/h using a syringe pump, and the solution was allowed to stir at room temperature for 12 h. The silica coated iron oxide nanoparticles were centrifuged, washed three times with ethanol and redispersed in MilliQ water.

### Materials characterisation

The morphology and size of Mag and Mag@SiO2 nanoparticles was characterized using JEOL 2010 high resolution transmission electron (HRTEM) microscope operated at an accelerating voltage of 200 kV. Samples for HRTEM measurements were prepared by drop casting particles on to a carbon-coated copper grid, followed by air drying. The crystallography of the nanomaterial powders was obtained on a Bruker D8 ADVANCE X-ray diffractometer using Cu Kα radiation. For magnetic measurements, a superconducting quantum interface device based magnetometer (Quantum Design MPMS-XL5) was used. The iron content of the nanoparticle solutions used for *in vitro* and *in vivo* studies was ascertained on a Varian AA280FS Fast Sequential Atomic Absorption Spectrometer (AAS) after digestion of particles overnight in nitric acid.

### *In vitro* cell studies and cytotoxicity assays

Human prostate cancer cells (PC3 cell line) were routinely cultured at 37°C in a humidified atmosphere with 5% CO2 using RPMI 1640 medium supplemented with 10% fetal bovine serum (FBS), 1% penicillin, 1% streptomycin/penicillin and 1 mM L-glutamine. For sub-culturing, PC3 prostate cancer cells were detached by washing with phosphate buffered saline (PBS) and incubating with trypsin-EDTA solution (0.25% trypsin, 1 mM EDTA) for 5 min at 37°C, followed by washing and incubation with supplemented RPMI 1641 medium. For cell uptake, the cells were first seeded in 24-well polystyrene dishes for 24 h, followed by incubation with Mag and Mag@SiO2 nanoparticles for 24 h at 37°C in complete cell media, and subsequent three times washing of cells with PBS, before imaging under an inverted microscope. For cytotoxicity assays, the viability of PC3 prostate cancer cells exposed to Mag@SiO2 nanoparticles in the absence of cell growth medium was determined. A CellTiter 96 AQueous One Solution Cell Proliferation Assay (Promega) kit containing the tetrazolium compound 3-(4,5-dimethylthiazol-2-yl)-5-(3-carboxymethoxyphenyl)-2-(4-sulfophenyl)-2H-tetrazolium (MTS), was used to monitor cell viability according to the manufacturer's protocols. MTS color change was monitored using a plate reader at 490 nm, and cell viability data was plotted by considering the viability for the untreated cells as 100%. Experiments were performed in triplicates, and error bars represent standard experimental errors.

### Magnetic resonance imaging (MRI) studies

MRI studies were performed for nanoparticle solutions stored in phantoms, in PC3 prostate cancer cells after nanoparticle uptake, and in a mouse model with breast cancer. For phantom MRI studies, phantoms were prepared in Eppendorf tubes with Mag@SiO2 nanoparticles at three different Fe concentrations (0.18 mM, 0.9 mM, 1.79 mM) and a saline solution without any nanoparticles was used as a control. For *in vitro* MRI studies, PC3 cancer cells were cultured using the above protocol in 24 well polystyrene plates, and incubated for 24 h with Mag and Mag@SiO2 nanoparticles at three different concentrations (0.18 mM, 0.9 mM, 1.79 mM) and a control with cells but no nanoparticles. MRI measurements for phantoms and PC3 cells were performed with a clinical 3.0 Tesla Clinical Siemens Trio MRI scanner using a 12-channel head coil and the following parameters: T2-weighted imaging, gradient echo sequence, multiple echo time (TE) ranging from 0.99–100 ms, repetition time (TR) = 2000 ms, matrix 128×128, slice thickness of 3 mm. Relaxation rates (R2) were determined by using a single echo sequence (SE) with a constant TR of 2000 ms and multiple TE ranging from 0.99–100 ms. The signal was plotted as a function of echo time and fitted to obtain the R2 values. The R2 values of the Mag@SiO2 in phantoms and PC3 cells were determined by plotting the relaxivity at a TE of 10.86 ms, as a function of molar iron concentration in respective samples, and extracting the T2 value from the slope by linear regression of data points obtained at lower Fe concentration values. Only lower Fe concentrations were used to determine the T2 values, predominantly because with increasing Fe concentrations above a particular threshold, the MR signals tend to loose their linearity. For the *in vitro* MRI measurements in phantoms and PC3 cells, enhancement of the R2 signal within the PC3 cells was calculated by: ΔR2/R2control*100. For *in vivo* MRI experiments, breast tumor bearing mice were developed in-house, anaesthetised with ketamine (80 mg per kg body weight) and xylazine (5 mg per kg body weight), and placed within the 12-channel head coil. Images were acquired before and after injection of 100 µL of Mag@SiO2 particles suspension of 100 µg/mL concentration in saline locally at the tumor site. A T2-weighted spin echo sequence was acquired with TE/TR of 60/2000 ms, a slice thickness of 3 mm and a 128x128 matrix. Data analysis was performed manually by placing ROIs in tumor and tissue areas on the images.
